# Comparative Evaluation of Microleakage in Endodontically Treated Teeth Using an Alkasite Restorative Material Versus Resin-Modified Glass Ionomer Cement (RMGIC) as an Intraorifice Barrier: A Study Protocol

**DOI:** 10.7759/cureus.70002

**Published:** 2024-09-23

**Authors:** Namrata P Jidewar, Manoj Chandak, Aditya Patel, Anuja Ikhar, Tejas Suryawanshi, Saee Wazurkar

**Affiliations:** 1 Department of Conservative Dentistry and Endodontics, Sharad Pawar Dental College and Hospital, Datta Meghe Institute of Higher Education and Research, Wardha, IND

**Keywords:** cention n, endodontics, intraorifice barrier, post-endodontic restoration, rmgic

## Abstract

Background

The most popular approach to treating pulpal and periapical disease is endodontic therapy, but the most common failure after root canal therapy, if not followed by planned rehabilitation of the tooth, is tooth fracture, which may be so severe in some cases needing extraction. Inadequate endodontic restorations increase the risk of fracture and coronal leakage, which can disseminate bacteria and their byproducts and perhaps lead to the failure of root canal therapy. In order to offer protection against masticatory forces along with compressive obturation forces that can lead to vertical root fracture, research has also supported the use of post-endodontic treatment restorative materials with an elastic modulus that is either greater or comparable to the tooth itself for post-endodontic restorations. Restorative materials that can bond to root dentin can be used to create intraorifice barriers, which prevent coronal microleakage and reinforce the radicular dentin. The current in vitro study aims to assess the effects of various intraorifice barriers (an alkasite restorative material versus resin-modified glass ionomer cement) on the sealing ability of the intraorifice barriers following root canal therapy.

Methodology

In vitro, confocal laser microscopy is used to determine the microleakage of various intraorifice barriers.

Conclusion

The sealing ability of a post-endodontic restoration is crucial for the overall success of endodontic treatment, and this in vitro study protocol would ultimately help in selecting the appropriate intraorifice barrier.

## Introduction

Endodontic therapy is the primary treatment option for periapical and pulpal diseases. However, several studies have shown that between 13% and 15% of all teeth that require extraction after endodontic therapy have cracks, craze lines, and vertical root fractures. It is widely recognized that teeth with insufficient post-endodontic restorations are susceptible to coronal leakage and fracture, which can result in the spread of bacteria and bacterial products and the potential failure of root canal therapy [1]. During chemomechanical preparation, medications and irrigating solutions alter the collagen structure, which profoundly alters the mechanical properties of dentin and speeds up the progression of fatigue fractures, raising the possibility of vertical root fracture [2]. In order to offer stiffness against forces that could contribute to a root fracture, research has also supported the use of restoration materials for root-filled teeth with an elastic modulus that is either higher than or comparable to the tooth either chemically or micromechanically.

Intraorifice barriers composed of materials that can adhere to radicular dentin could be used to stop coronal microleakage, and material bond proprieties would strengthen the dentin surface. Although intraorifice barriers have been compared extensively in the literature on their fracture resistance, there is a dearth of research discussing the sealing ability of teeth reinforced with the materials utilized in the study when placed inside a root canal as post-endodontic reinforcement material [3,4]. Inadequate post-endodontic restorations raise the possibility of coronal leakage, which might let microbes and their metabolites in and perhaps cause root canal therapy to fail. Consequently, two objectives of post-endodontic restoration are to increase root fracture resistance and create an impermeable hermetic seal [4]. Intraorifice barriers can be used to stop coronal microleakage. Thus, the present study aims to assess how various intraorifice barriers (such as Cention N and glass ionomer cement mixed with resin) impact the ability of the teeth to close following root canal therapy. This is an in vitro study on human mandibular premolars as per the institutional ethical clearance committee of DMIHER.

## Materials and methods

Estimation of sample size

The type I error rate is established at a significance level of α = 0.05. The Z alpha value is 90%. Power (1-beta) = 0.8 1.645; 80% is the Z beta value. The sample size ratio is 0.842, with a treatment/control ratio of 1. Permissible variation, d=μT− μC= 0.3 δ(>0), margin = 1.1. The expected population standard deviation (SD) is 0.9. Drop rate (%) = 9. Groups 1 and 2 each consist of 10 samples per group, resulting in a total of 20 samples. Based on the aforementioned computation, the sample size was estimated to be 10 samples per group after accounting for dropouts. A total of 20 samples have been established.

Sample selection 

Inclusion Criteria

The study included noncarious, extracted mandibular first premolar teeth with a curvature of less than 10°. The dimensions of the coronal aspect will be measured in both buccolingual and mesiodistal directions, and variations up to 10% will be accepted. The sample will be defined as a variation from these mean values obtained by up to ± 10%.

Exclusion Criteria

Teeth examined with a stereomicroscope at 10× magnification for the purpose of looking for cracks or craze lines will be discarded. Teeth having multiple canals, cavities, open apices, and root curvature greater than 10° will be disqualified. All the samples will be subjected to radiographic evaluation to confirm that they have a single canal. Teeth with internal or external resorption will not be accepted and will be stored in 0.1% thymol solution until used in the study.

Materials required

This study utilized glass ionomer cement modified by resin, also known as Fuji PLUS RMGIC (resin-modified glass ionomer cement) (GC International, Houston, Texas) (Group 1) and Cention N (a restorative alkasite group substance) (Viva Dent Ivoclar, Schaan, Liechtenstein) (Group 2).

Sample preparation and method

For this study, 20 whole mandibular first premolars with a single canal will be selected for several reasons, including sample processing. The samples will be decoronated to a standardized 14 mm length using a diamond disc and water coolant. Using the #10K file, working length and patency will be determined. A rotary ProTaper Universal system (Dentsply, Charlotte, North Carolina) will be used to instrument the canals utilizing the crown-down procedure until F3. After every file change, canals will be irrigated with 2 mL of 5% sodium hypochlorite and distilled water during instrumentation and final irrigation with 5 mL of 17% EDTA. A warm vertical compaction method with matching gutta-percha points (Dia-ProT Gutta Percha) and a resin-based sealer (diadent dia-proseal, DiaDent Group International, South Korea) will be used for obturation. To place intraorifice barriers, the samples will be randomly distributed to two groups (n = 10). All specimens, with the exception of the control group, will be processed using a heating instrument to remove the coronal 3 mm of gutta-percha.

For Group 1, alkasite restorative material (Cention N), powder and liquid components, will be combined per the manufacturer's directions and placed into the cavity. It will undergo a 30-second light treatment. For Group 2, the primer for RMGIC (Vitremer) dentin will be applied with a microtip and light-cured for approximately 20 seconds. Following the manufacturer's recommendations for mixing, the material will be placed into the cavity, allowed to condense, and then exposed to light for 40 seconds. All specimens will be kept in an incubator for one week at 37°C and 100% humidity following the insertion of intraorifice barrier materials. After the varnish application, the roots will be exposed to a fluorescent dye (rhodamine B, 0.1%) in the form of an aqueous solution for 24 hours. After that, it will be further cut into a cross-section, and the amount of dye penetration will be measured using a confocal microscope. CLSM (confocal laser scanning microscopy, 10× magnification) will be used to evaluate each region. A wavelength of 561 nm will be used for emission. The maximal dye penetration depths (μm) in the dentinal tubules will be ascertained by processing digital photographs at four peripheral points: 12, 3, 6, and 9 o'clock. This will be done using the Image J (ImageJ application, NIH) software. The tool's "distance" will be applied from the root canal surface to the deepest point of the visible dye [4].

## Results

Since this is a protocol for an in vitro study, the results have yet to be obtained. The final results will be established after the study is completed. The null hypothesis for the present protocol would be that there is no difference in the sealing ability between the different intraorifice barriers.

## Discussion

Thanks to root canal therapy, dentists can now salvage teeth that would have been immediately pulled a few decades ago. Conversely, teeth receiving endodontic treatment are assumed to be more likely to fracture than healthy teeth. Following endodontic therapy, it is imperative to restore and safeguard the residual tooth structure [5]. The significance of procedures carried out subsequent to endodontic therapy for the outcome of non-vital teeth has drawn more attention. However, there is no discernible variation in the moisture content of teeth during endodontic treatment compared to healthy teeth [6]. Because force applied to the tooth's long axis transmits equally, as demonstrated by several experiments, the force will also be delivered vertically along the tooth's long axis in this study [7]. The materials employed in this study will be compared as intraorifice barrier materials because they have characteristics such as high strength and an elasticity modulus similar to those of dentin.

Introduced in the late 1980s, RMGIC includes some methacrylate components present in resin composites. Due to the improved performance of "RMGIC," which is explained by the material's "water sorption," Tselnik et al. observed greater performance as a satisfactory coronal seal [8]. Setting expansion causes a better seal to be achieved. It can cling to dentin and does not need to be pretreated.

The release of fluoride is another beneficial characteristic of RMGIC. RMGIC is a material that can tolerate high stress levels before passing the load to the root because of its high flexural strength and modulus of elasticity, comparable to those of dentin [8-10]. The elasticity modulus of centroid N is 13 GPa. Additionally, it contains a proprietary isofiller that relieves shrinkage stress and aids in the recovery of polymerization shrinkage. It also forms a micromechanical bond with the tooth structure. Because filler particles are nanoparticle-sized, isofiller increases microhardness. It aids in withstanding the strains and tensions placed on the mouth mucosa. It can also be positioned cautiously, strengthening the tooth structure that is still present [11,12].

## Conclusions

This study will assist endodontists and general dentists in selecting the best and most effective filling materials to utilize as an intraorifice barrier, increasing the sealing ability of the teeth that have undergone endodontic treatment. Modern dental materials will enable a more comprehensive view of the application of an intraoral barrier, increasing the teeth's capacity to seal endodontically treated areas.
